# Homing Pigeons Only Navigate in Air with Intact Environmental Odours: A Test of the Olfactory Activation Hypothesis with GPS Data Loggers

**DOI:** 10.1371/journal.pone.0022385

**Published:** 2011-08-03

**Authors:** Anna Gagliardo, Paolo Ioalè, Caterina Filannino, Martin Wikelski

**Affiliations:** 1 Department of Biology, University of Pisa, Pisa, Italy; 2 Department for Migration and Immuno-ecology, Max Planck Institute for Ornithology, Radolfzell, Germany; Università di Parma, Italy

## Abstract

A large body of evidence has shown that anosmic pigeons are impaired in their navigation. However, the role of odours in navigation is still subject to debate. While according to the *olfactory navigation hypothesis* homing pigeons possess a navigational map based on the distribution of environmental odours, the *olfactory activation hypothesis* proposes that odour perception is only needed to activate a navigational mechanism based on cues of another nature. Here we tested experimentally whether the perception of artificial odours is sufficient to allow pigeons to navigate, as expected from the olfactory activation hypothesis. We transported three groups of pigeons in air-tight containers to release sites 53 and 61 km from home in three different olfactory conditions. The Control group received natural environmental air; both the Pure Air and the Artificial Odour groups received pure air filtered through an active charcoal filter. Only the Artificial Odour group received additional puffs of artificial odours until release. We then released pigeons while recording their tracks with 1 Hz GPS data loggers. We also followed non-homing pigeons using an aerial data readout to a Cessna plane, allowing, for the first time, the tracking of non-homing homing pigeons. Within the first hour after release, the pigeons in both the Artificial Odour and the Pure Air group (receiving no environmental odours) showed impaired navigational performances at each release site. Our data provide evidence against an activation role of odours in navigation, and document that pigeons only navigate well when they perceive environmental odours.

## Introduction

Almost four decades ago, Papi and colleagues [1] showed that homing pigeons without the ability to smell have dramatically reduced navigational abilities. Since then, a large body of evidence has been collected showing that lesions to the olfactory system consistently produce a disruption of the birds' homing abilities (see for ref [2]–[5]). This phenomenon has been explained by Papi with the olfactory navigation hypothesis [6], which predicts that pigeons are able to build up an odour-based navigational map by associating the wind-borne environmental odours with the directions from which they blow at the home area. Once displaced, the pigeons are able to recognise the local prevalent odours characterising the release location, thereby determining the direction of displacement. Once the birds know where they are relative to home, they can orient by using a sun or a magnetic compass. Thus in its most general form, the olfactory navigation hypothesis proposes that in pigeons environmental odours are integrated to build up a mental representation of wide geographical areas around the home loft.

However, the olfactory navigation hypothesis is not uncontested [7]. Recently, Jorge and colleagues [8], [9] suggested that impaired navigation of anosmic pigeons is due to the fact that olfactory stimuli prime the navigational capabilities of birds. Under this hypothesis, environmental odours are solely needed to activate a navigational system that in turn is based on non-olfactory cues [8], [9]. In support of this hypothesis the authors reported that pigeons transported in charcoal-filtered air and released after anaesthesia of the olfactory mucosa displayed scattered initial orientation. In contrast, pigeons transported in pure air, but stimulated with artificial non-sense odours, were not different from control pigeons, irrespective of the fact that the nasal anaesthesia before release prevented them from smelling environmental odours.

However, it is difficult to reconcile the above findings [8], [9] with a large body of previous evidence against a priming role of odours on a non-olfactory navigational system. In particular, experiments testing intact pigeons after manipulations of their housing conditions during map learning contradict the odour-priming hypothesis. Pigeons were unable to develop navigational abilities if raised in aviaries provided with screens preventing the birds from detecting wind directions [10]–[12]; in contrast, pigeons exposed to the natural winds were able to orient, even if the view of the surroundings was obstructed [13]. When the directions of the winds at the home loft were deflected [14]–[16] or inverted [17], a correspondent deflection or inversion of the birds' initial orientation was observed. Other important evidence that odours provide spatial information useful for navigation comes from experiments in which pigeons exposed to artificial odour currents during map learning displayed the expected orientation on the basis of the odour stimuli provided at the release site [18], [19]. It is worth noting that artificial odour stimuli at the release site determined the expected orientation only if they had been associated by the birds with the artificial wind direction during map learning [18], [19], while they acted as disturbing factors if they just represented non-sense odours [20], [21]. Other important evidence, supporting a specific role of environmental odour cues in navigation comes from an experiment in which pigeons, transported in air-tight containers ventilated with pure air, were exposed at the release site to air sampled either from an open field or from a thick vegetation area (maize field or forest) before being released after nasal anaesthesia [22]. This experiment showed that the birds exposed to air of the release site sampled from the thick vegetation area were poorer in homeward orientation than the birds allowed to smell the release site air collected in an open field, probably more representative of the specific odour profile of the release site. All these findings are not explained by assuming a priming role of odours in pigeon navigation, but only by accepting a specific role of environmental smells in the proposed navigational map.

So far, the conflicting evidence supporting either a specific role or a priming role of olfactory stimuli in pigeon navigation has come from experiments reporting only the initial orientation data achieved by recording vanishing bearings. With the present experiment we achieve a major advance in our understanding of the role of olfactory stimuli in pigeon navigation by being able to additionally study homing pigeons' tracks even when the birds do not home. We used newly developed 1 Hz-GPS data loggers that allow for a remote readout of the stored data via a 900 MHz data link [23], sending data for up to 18 km from the surface to a small Cessna plane in the air.

To conduct this experiment we transported the pigeons, and kept them at the release site, in air-tight containers where they were exposed to three different odour stimuli conditions. Birds were then released after anaesthesia of their olfactory mucosa. One group of birds was allowed to breath environmental air, one group was exposed only to pure air and a third group was exposed to pure air, but stimulated with artificial non-sense odours. From these treatments we derive two exclusive predictions: i) The olfactory navigation hypothesis is rejected, the olfactory priming hypothesis is accepted: under this scenario both control pigeons and those exposed to ‘priming’ odours will navigate equally well. We thus predict unimpaired navigational performances in both of these groups, but not the group that lacks exposure to any environmental odours during transport and before release. Alternatively, ii) the olfactory navigation hypothesis is accepted and the olfactory priming hypothesis is rejected: here we predict that only the control birds show unimpaired navigational abilities while both other groups that are deprived of environmental odours are impaired. Our data support the latter scenario and thus provide evidence for the olfactory navigation hypothesis.

## Materials and Methods

### General procedure

Thirty-six inexperienced pigeons, about 15–18 months of age and hatched at the Arnino field station (latitude 43° 39′ 26′′ N; longitude 10° 18′ 14′′ E), Pisa, Italy, were used in the study. The pigeons were raised as free flyers and were kept and manipulated according to Italian law on animal welfare. Our experiment has been conducted in accordance with the Italian law and has been approved by the Ethical Committee of the University of Pisa (C.A.S.A.), with permit number 0005799 22/11/2010. Twenty days prior to the experimental releases all the birds were equipped with a PVC dummy weight, similar in dimension and weight to the GPS data logger they would be carrying, in order to accustom them to flying with a load. The dummy was attached to the pigeons' back by means of a Velcro strip glued on the feathers, which had been trimmed. A few days before the experimental tests, the pigeons had been released in a group from different directions up to 7 km from Arnino.

For the test releases we transported three groups of pigeons and kept them at the release site in air tight containers ventilated by aspirators. The Control (C) group container was ventilated by environmental air; both the Pure Air (PA) group and the Artificial Odour (AO) group containers were ventilated by pure air filtered through an active charcoal filter; in addition the Artificial Odour group received different puffs of odours of plant origin (eucalyptus, orange, jasmine, rose, lavender) both during transportation and at the release site about each 20 minutes for the duration of the whole experiment. The administration of the odour puffs was given injecting a 50 ml volume of air saturated with one odour, in the flux of air coming from the charcoal filter to the air tight container. The release experiment started two hours after we had arrived at the release site.

### GPS data loggers

We used two different kinds of miniature GPS data loggers storing one position fix every second: for twenty of the tests on C birds we used the loggers by Technosmart (www.technosmart.eu, Rome, Italy); for the other release tests we used loggers by E-obs (www.e-obs.de, Munich, Germany), which also feature remote UHF data download capabilities. The latter device thus allowed aerial data downloads (from a small Cessna airplane, the ‘Spirit of MaxCine’) of pigeons that did not home. The positional fixes stored by a GPS data logger include latitude, longitude, and time of recording. The tracks for each pigeon were uploaded, automatically checked for potential GPS-errors and duplicates, and then stored and made publicly accessible in Movebank [24] (www.movebank.org) for each recorded release. Data were then exported from Movebank and were visualised with Google Earth.

### Test releases

Eleven C, 8 PA pigeons and 8 AO birds were released from Bolgheri (Long. 10° 34′ 35′′ Lat. 43° 13′ 07′′; home direction 337°, home distance 53 km). Eleven C pigeons, 7 PA birds and 7 AO pigeons were released at Montespertoli (Long. 11° 03′ 59′′ Lat. 43° 39′ 09′′; home direction 270°, home distance 61 km). Before the release each pigeon was subjected to anaesthesia of the olfactory mucosa with a single dose of Xylocaine® sprayed through the choanae and then equipped with a GPS data logger. Each pigeon was released singly. The releases took place under sunny conditions, with no or light wind. The homing time on the day of the release was recorded by an observer at the home loft.

### Quantitative analyses and statistical procedures

Because the effect of the local anaesthesia of the olfactory mucosa decreases over time [25], we analysed separately the sections of the tracks from take-off until one, two and three hours after release, respectively. For the analysis we considered the directions taken by the bird while moving from one point to the next at a speed higher than 5 km/h. We thus calculated the individual mean vector and the relative homeward component. The mean vector distributions relative to the section of the tracks recorded in the first hour, in the first two hours and three hours of each experimental group at both sites were tested for randomness with the one sample Hotelling test [26]. We also calculated the homeward component (hc) of the second order mean vectors.

Focusing on the first hour after release, we performed a statistical analysis by pooling the data from the two release sites. We considered all tracks of the birds released once, plus the first track suitable for the analysis of the birds released twice. We assessed the performances of the three experimental groups by considering the following parameters, which have been compared with the Kruskall Wallis test: the tortuosity of the track expressed by the individual mean vector length, the orientation of the track expressed by the homeward component of the mean vector, and the efficiency index of the flight. The efficiency index for each pigeon is defined as the beeline between the release site and the point where the bird was after 1 hour from release, divided by the length of the flight path.

Furthermore, to enable comparisons with previously published data, we recorded the virtual vanishing bearings, defined as the directions with respect to the release site of the birds at a distance of 2 km from it. If a pigeon did nothing but circle around the release site at a distance closer than 2 km, it was excluded from the analysis. The circular distributions were tested for randomness by means of both the Rayleigh and V test and compared with the Mardia-Watson-Wheeler test [26].

As the birds sometimes stopped (i.e., either landed or moved at a speed lower than 5 km/h) during their homing journey, we calculated the percentage of time the bird was flying in the track recorded in the first hour after release. In addition, we calculated how far the birds were from home at the end of their first hour track section and we recorded the birds homing performances. We compared the percentage of time in flight, the distances at which the birds were after 1 hour and the homing performance of the three groups with the Kruskall-Wallis test. All pigeons we released were included in the analysis of the homing performances. The Dunn's test was used for multiple comparisons.

## Results

Some of the Technosmart GPS data loggers used on control pigeons did not record or only started to record a few hours after release and were therefore excluded from the analysis on orientation. For this reason we had to include some additional birds in the control group. Thus the number of pigeons belonging to the C groups was higher, but we obtained a lower number of tracks suitable for analysis.

**Figure 1:**
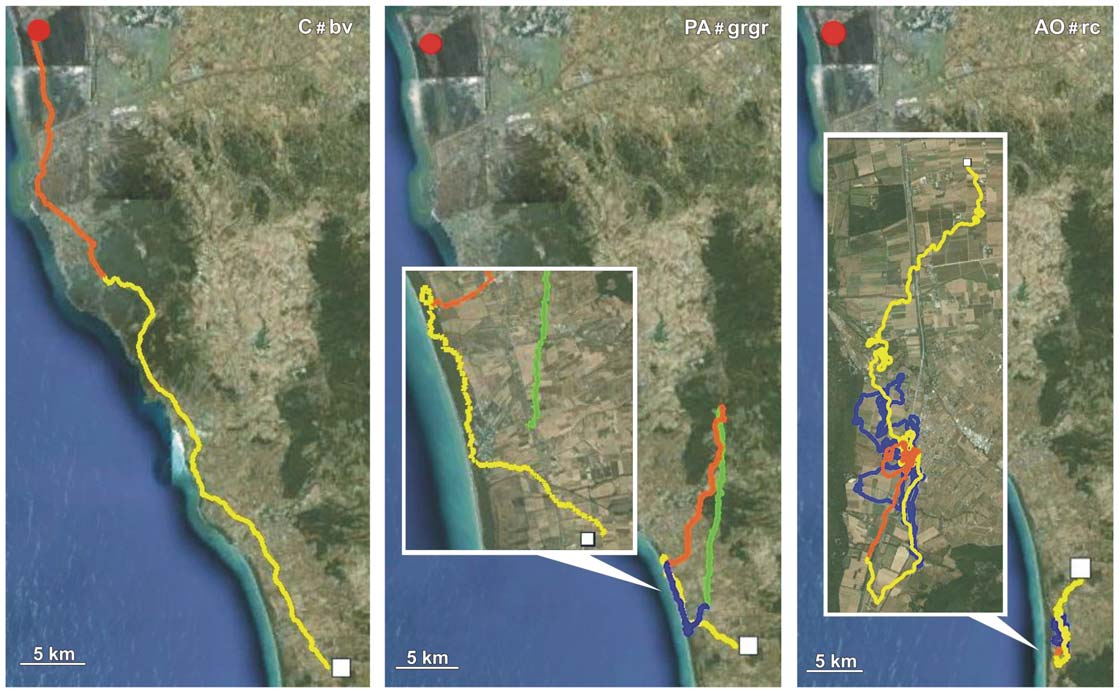
Examples of tracks: the code of the track and the experimental group is reported on each figure. The red circle and white square represent home and release sites (Bolgheri South and Montespertoli East from home), respectively. The yellow, red, green and blue lines: paths flown during the first, second, third and subsequent hours from release. Triangles indicate that the bird stopped for an entire hour. The colour of the triangle indicates the time range in which the bird stopped.

**Figure 2:**
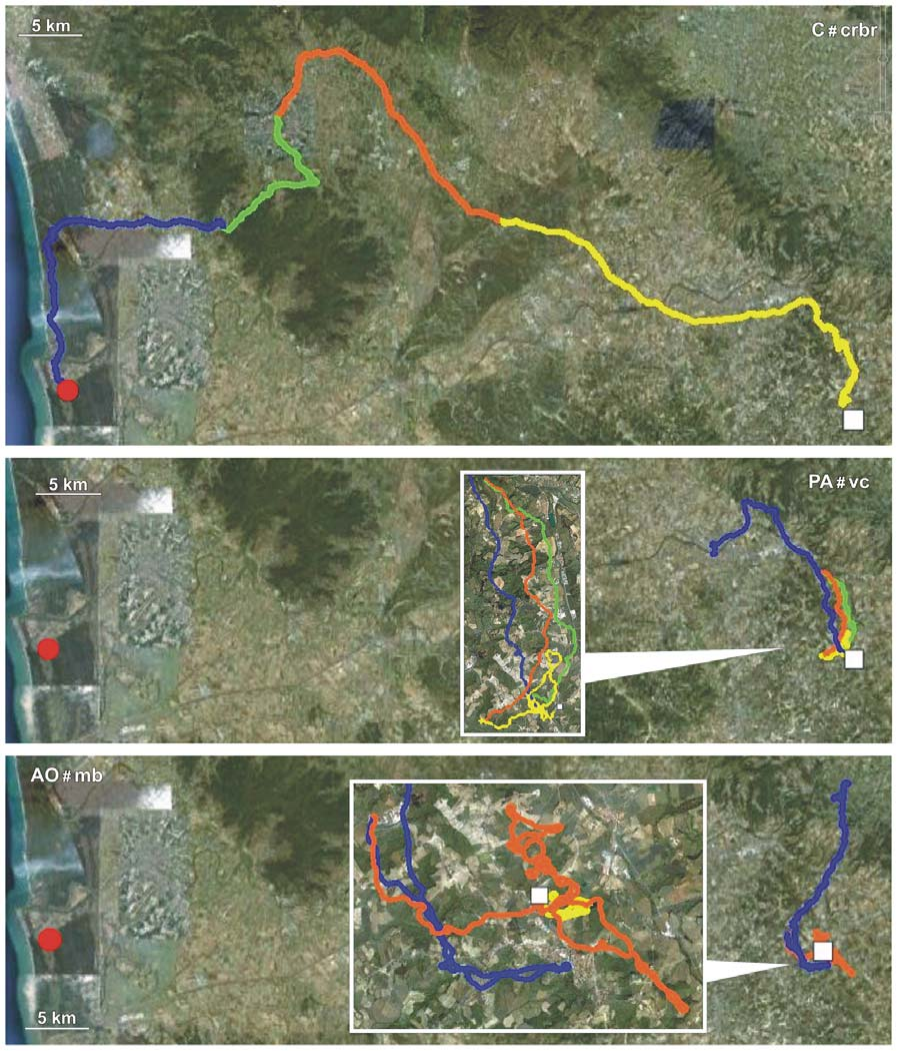
Examples of tracks. Other explanations as in Figure 1.

**Figure 3:**
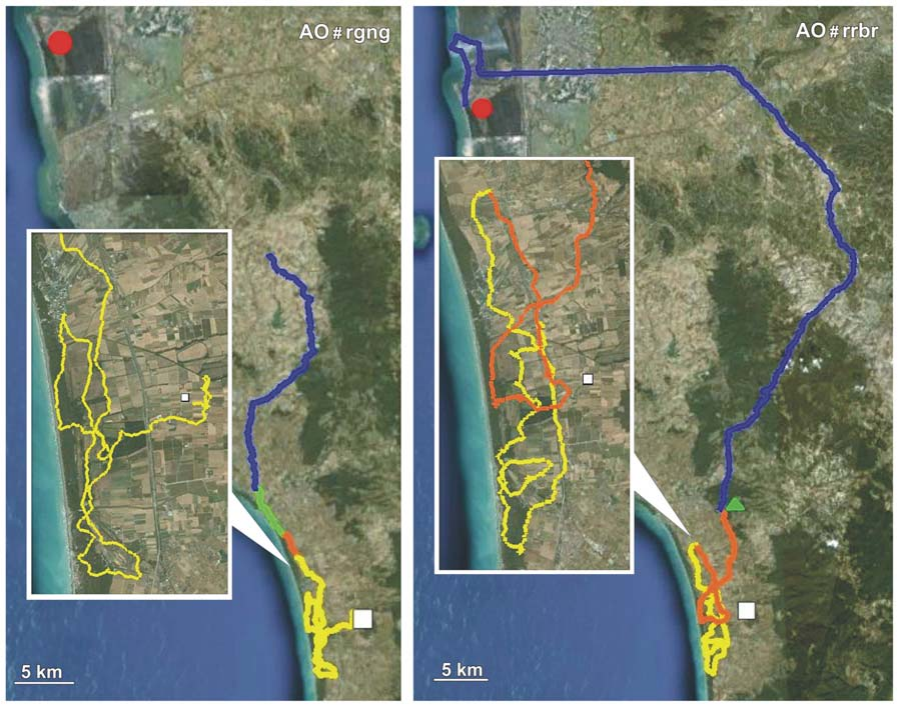
Examples of tracks. Other explanations as in Figure 1.

**Figure 4:**
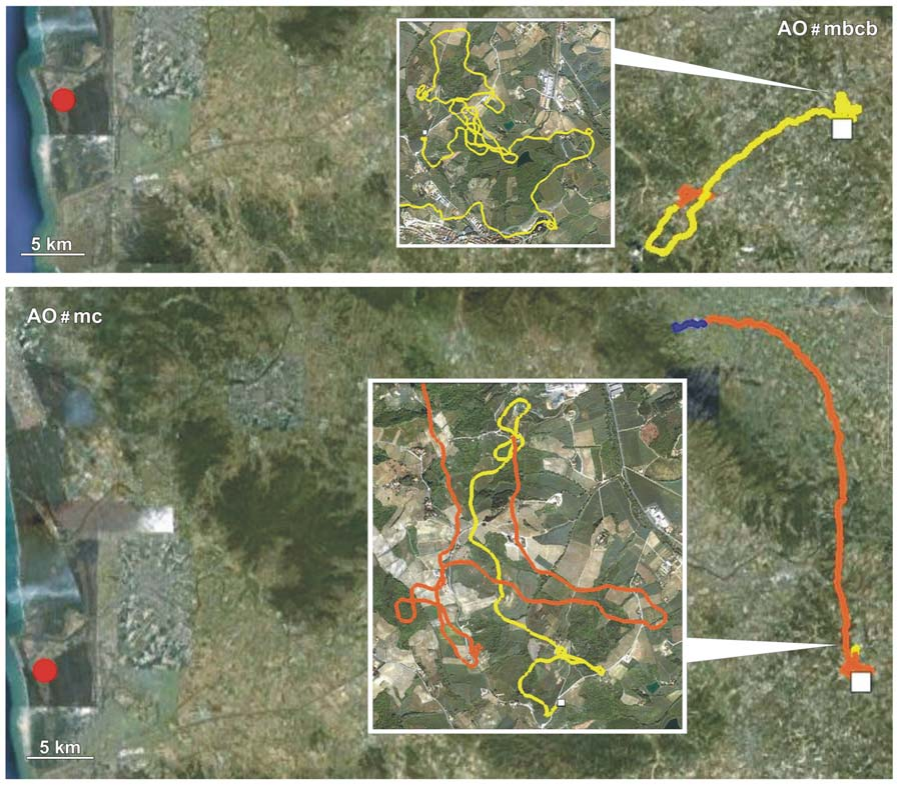
Examples of tracks. Other explanations as in Figure 1.

**Figure 5:**
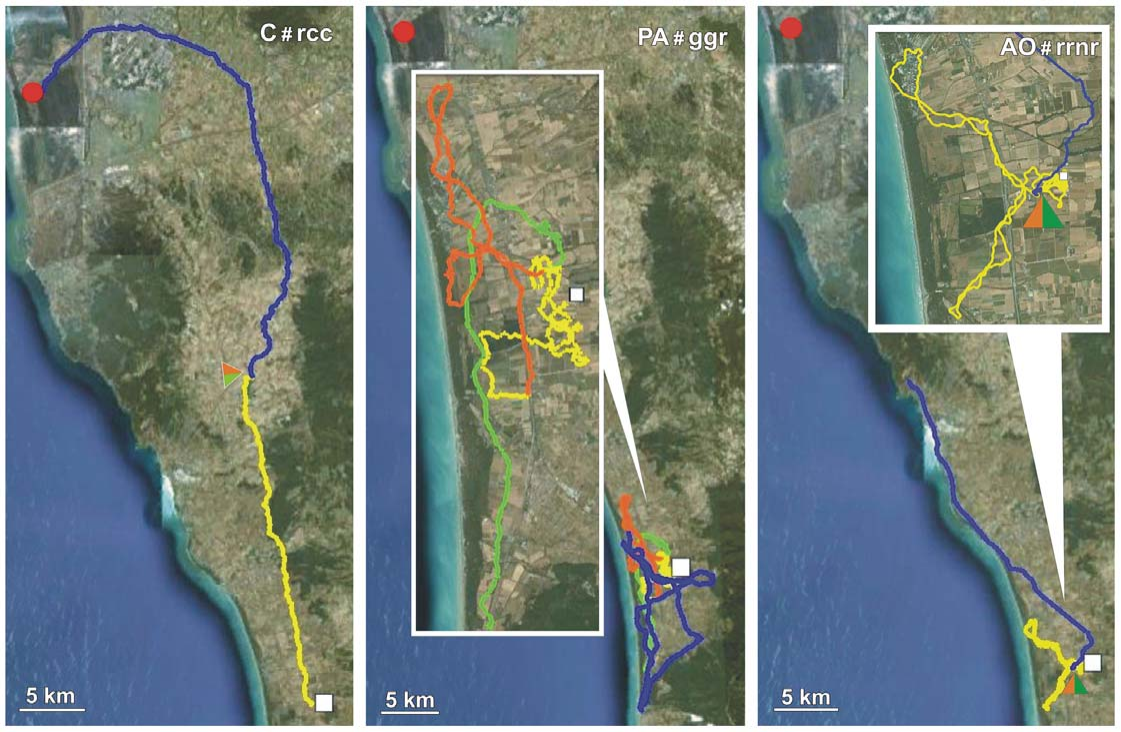
Examples of tracks. Other explanations as in Figure 1.

**Figure 6:**
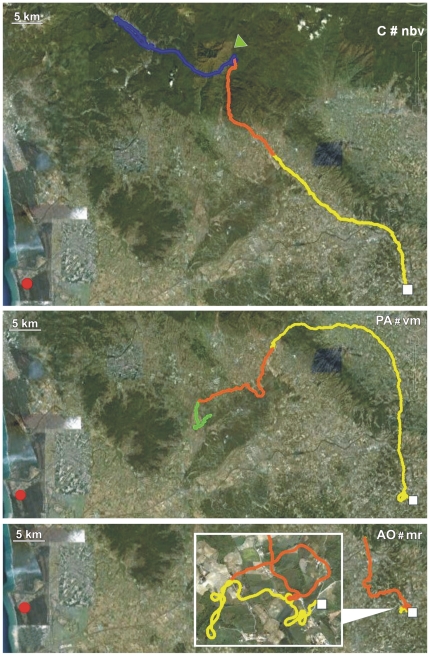
Examples of tracks. Other explanations as in Figure 1.

Details on the performances of Control, PA and AO pigeons obtained in both the release from Bolgheri (North) and Montespertoli (East) are reported in Table 1, 2 and 3, respectively. Examples of tracks of Controls, PA and AO are visualised in Figures 1, 2, 3, 4, 5, and 6, which show the portion of the route flown during the first (yellow), the second (red), the third (green), and following hours (blue). All the tracks can be inspected in Movebank (www.movebank.org).

**Table 1:** Control Pigeons.

RS	track	Date	r (1)	α (1)	r (2)	α (2)	r (3)	α (3)	ht	at
Bolgheri 336° 53 km	#bb^1^	02/07/10	0.56	321°	0.51	334°	0.66	351°	7^h^ 55′	A, P
	#bc^2^	02/07/10	0.79	331°	0.31	350°	0.28	351°	day after	A, P
	#bv^3^	02/07/10	0.83	332°	0.83	336°	0.83	336°	2^h^ 15′	A, P
	#bgbg^4^	05/07/10	-	-	-	-	-	-	day after	NR
	#brgr^5^	05/07/10	-	-	-	-	-	-	6^h^ 25′	I
	#br^6^	05/07/10	-	-	-	-	-	-	8^h^ 45′	I
	#rmb^7^	10/07/10	0.42	333°	0.68	331°	0.69	332°	2^h^ 42′	A
	#rcb^8^	10/07/10	0.25	303°	0.17	300°	0.11	297°	6^h^ 02′	A
	#rcc^9^	10/07/10	0.71	347°	0.70	347°	0.55	351°	6^h^ 39′	A
	#rvc^10^	10/07/10	-	-	-	-	-	-	day after	I
	#rvgr^11^	10/07/10	0.69	336°	0.48	346°	0.45	346°	4^h^ 35′	A
Montespertoli 270° 61 km	#cgr^12^	07/07/10	0.75	325°	0.75	325°	0.75	325°	lost	A, P
	#cb^8^	07/07/10	0.40	231°	0.19	284°	0.23	305°	later	A, P
	#crbr^13^	07/07/10	0.64	299°	0.61	305°	0.48	305°	4^h^ 45′	A
	#cc^9^	07/07/10	0.63	187°	0.44	287°	0.40	288°	day after	A, P
	#cm^14^	07/07/10	-	-	-	-	-	-	lost	I
	#cr^6^	07/07/10	-	-	-	-	-	-	day after	NR
	#cbcb^15^	07/07/10	-	-	-	-	-	-	lost	NR
	#nbb^1^	21/07/10	-	-	-	-	-	-	9^h^ 16′	I
	#nrg^16^	21/07/10	-	-	-	-	-	-	6^h^ 00′	NR
	#nrgng^17^	21/07/10	-	-	-	-	-	-	day after	NR
	#nbv^3^	21/07/10	0.80	314°	0.76	321°	0.67	321°	day after	A

RS: release site, home direction and distance; Track: track code and individual pigeon code number are reported; Date: date of release; r and α: mean vector length and directions relative to the sections of tracks recorded in the first (1), the two first (2) and the three first (3) hours after release (see Material and methods for further explanations); ht: homing time expressed in hours and minutes; “day after”, the pigeon homed the day after the release, “later”, the bird homed in the subsequent days, “lost” the bird never homed; at, analysis of the tracks: A, track used in the single release statistical analysis and P used also in the pooled analysis; I, incomplete track which could not be used in the statistical analysis; NR the track was not recorded by the logger. Release from Bolgheri: five out of 11 C pigeons tested at Bolgheri had previously tested at Montespertoli (two former C, two former PA and one former AO). Release from Montespertoli: Six out of 11 C pigeons tested at Montespertoli had been previously released at Bolgheri (3 former C and 3 former AO).

**Table 2:** Pure Air Pigeons.

RS	track	Date	r (1)	α (1)	r (2)	α (2)	r (3)	α (3)	ht	at
Bolgheri 336° 53 km	#ggbg^18^	02/07/2010	0.44	334°	0.44	329°	0.46	331°	9^h^ 00′	A, P
	#ggr^19^	02/07/2010	0.12	213°	0.08	307°	0.14	212°	later	A, P
	#gm^20^	02/07/2010	0.42	347°	0.52	337°	0.54	335°	4^h^ 50′	A, P
	#gr^16^	02/07/2010	0.16	309°	0.15	311°	0.15	309°	later	A, P
	#grgr^21^	02/07/2010	0.64	322°	0.60	353°	0.13	305°	later	A, P
	#gb^22^	05/07/2010	0.66	116°	0.12	316°	0.11	314°	later	A, P
	#gv^23^	05/07/2010	0.53	331°	0.56	330°	0.22	332°	day after	A, P
	#gc^24^	05/07/2010	0.78	328°	0.74	335°	0.72	335°	day after	A, P
Montespertoli 270° 61 km	#vv^25^	7/07/2010	0.48	278°	0.47	277°	0.47	277°	day after	A, P
	#vrgr^5^	7/07/2010	0.14	136°	0.18	236°	0.18	236°	day after	A, P
	#vr^26^	7/07/2010	0.78	266°	0.77	266°	0.77	266°	8^h^ 35′	A, P
	#vm^20^	7/07/2010	0.52	317°	0.38	301°	0.31	301°	7^h^ 05′	A
	#vgr^11^	7/07/2010	0.74	312°	0.64	310°	0.64	310°	day after	A, P
	#vc^10^	7/07/2010	0.16	280°	0.37	348°	0.04	347°	day after	A, P
	#vbcb^27^	7/07/2010	0.27	332°	0.51	330°	0.66	303°	lost	A, P

Explanations as in Table 1. Two out of 7 PA pigeons tested at Montespertoli had been released at Bolgheri (1 former C and 1 former PA).

**Table 3:** Artificial Odour Pigeons.

RS	track	Date	r (1)	α (1)	r (2)	α (2)	r (3)	α (3)	ht	at
Bolgheri 336° 53 km	#rgrg^28^	02/07/2010	0.21	275°	0.38	357°	0.38	357°	day after	A, P
	#rrnr^29^	02/07/2010	0.07	275°	0.07	275°	0.07	275°	later	A, P
	#rrbr^13^	02/07/2010	0.20	324°	0.15	337°	0.15	337°	day after	A, P
	#rgng^17^	02/07/2010	0.27	316°	0.31	319°	0.37	321°	day after	A, P
	#rgcg^30^	02/07/2010	0.59	070°	0.58	073°	0.29	115°	lost	A, P
	#rc^31^	05/07/2010	0.26	193°	0.14	190°	0.14	190°	later	A, P
	#rb^32^	05/07/2010	0.06	191°	0.27	332°	0.26	334°	day after	A, P
	#rrnr2^33^	05/07/2010	0.04	113°	0.22	236°	0.38	326°	day after	A, P
Montespertoli 270° 61 km	#mv^34^	7/07/2010	0.34	302°	0.48	323°	0.43	299°	lost	A, P
	#mgr^19^	7/07/2010	0.25	351°	0.06	046°	0.26	217°	day after	A
	#mgbg^4^	7/07/2010	0.75	340°	0.48	284°	0.48	284°	5^h^ 00′	A, P
	#mc^2^	7/07/2010	0.44	352°	0.07	328°	0.59	336°	later	A, P
	#mbcb^35^	7/07/2010	0.34	242°	0.26	249°	0.26	249°	day after	A, P
	#mb^7^	7/07/2010	0.03	162°	0.13	306°	0.12	307°	later	A, P
	#mr^36^	7/07/2010	0.23	301°	0.45	333°	0.42	333°	lost	A, P

Explanations as in Table 1. Three out of 7 AO pigeons tested at Montespertoli had been previously used at Bolgheri (2 former C and 1 former PA).

**Figure 7:**
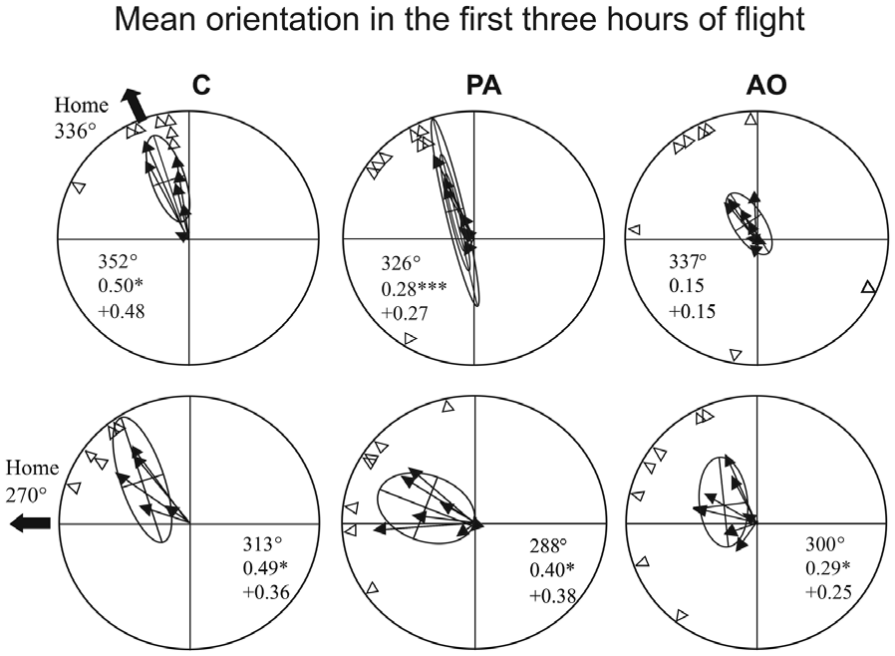
Mean vector distributions relative to the sections of the tracks recorded in the first three hours after release from Bolgheri (home direction 336°) and Montespertoli (home direction 270°), respectively. Outer arrows: home direction. Inner arrows: individual mean vectors (see Material and methods for further explanations). The open triangles at the periphery of the circle represent the directions of the individual mean vectors. Confidence ellipses of the distributions are reported. The second order mean vector lengths and directions are reported inside the circles. Asterisks indicate the significance level of the one sample Hotelling test: * p<0.05; ** p<0.01; *** p<0.001.

**Figure 8:**
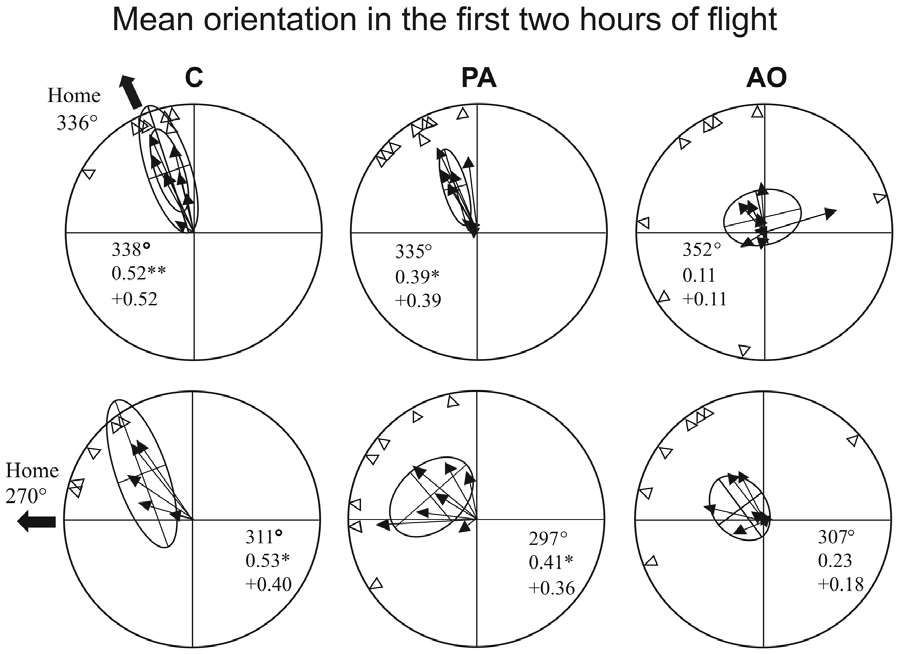
Mean vector distributions relative to the sections of the tracks recorded in the first two hours after release. Further explanations as in Figure 7.

**Figure 9:**
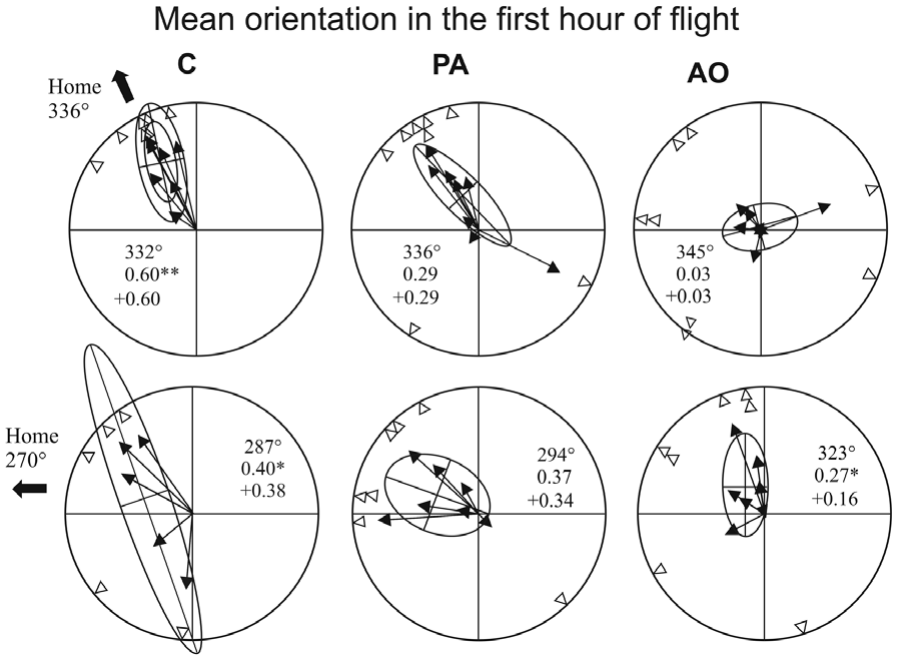
Mean vector distributions relative to the sections of the tracks recorded in the first hour after release. Further explanations as in Figure 7.

Considering the portions of the tracks recorded within the first three hours after release (Figure 7) we observed that the individual mean vector distributions were significantly different from random at both release sites for both C (Hotelling test, Bolgheri: p<0.05, T_(2,5)_ = 26.45435; Montespertoli: p<0.05 T_(2,3)_ = 72.19185) and PA (Hotelling test, Bolgheri: p<0.001, T_(2,6)_ = 98.762; Montespertoli: p<0.05 T_(2,5)_ = 15.9391). The AO pigeons' mean vector distribution is randomly scattered at Bolgheri (Hotelling test, p>0.05 T_(2,6)_ = 3.58884) and significantly different from uniform at Montespertoli (p<0.05 T_(2,5)_ = 27.68512). If we restrict the analysis to the portions of the tracks recorded within the first two hours after release (Figure 8) the pigeons' distributions of both C (Hotelling test, Bolgheri: p<0.01, T_(2,5)_ = 33.22055; Montespertoli: p<0.05 T_(2,3)_ = 55.34831) and PA (Hotelling test, Bolgheri: p<0.05, T_(2,6)_ = 24.73326; Montespertoli: p<0.05 T_(2,5)_ = 30.00647) are still significantly oriented at both sites. By contrast, the AO birds' mean vector distributions are not significantly different from random at both sites (Hotelling test, Bolgheri: p>0.05 T_(2,6)_ = 3.268577; Montespertoli: p>0.05 T_(2,5)_ = 9.930811). The observation of the behaviour of the birds within the first hour after release (Figure 9) revealed that the C birds displayed mean vector distributions different from random at both sites (Hotelling test, Bolgheri: p<0.01, T_(2,5)_ = 71.43776; Montespertoli: p<0.05 T_(2,3)_ = 68.66537), the PA birds were randomly scattered at both sites (Hotelling test, Bolgheri: p>0.05, T_(2,6)_ = 8.58531; Montespertoli: p>0.05 T_(2,5)_ = 10.38986), and the AO pigeons turned out to be randomly scattered at Bolgheri (Hotelling test, p>0.05 T_(2,6)_ = 0.4150136); and significantly oriented at Montespertoli (Hotelling test, p<0.05 T_(2,5)_ = 14.82878). However, the AO birds, although significantly oriented, showed a very small homeward component (see Figure 9 for details).

As the nasal anaesthesia decreases over time, a detailed analysis of the section of the tracks recorded in the first hour after release is reported below.

### Efficiency index

**Figure 10:**
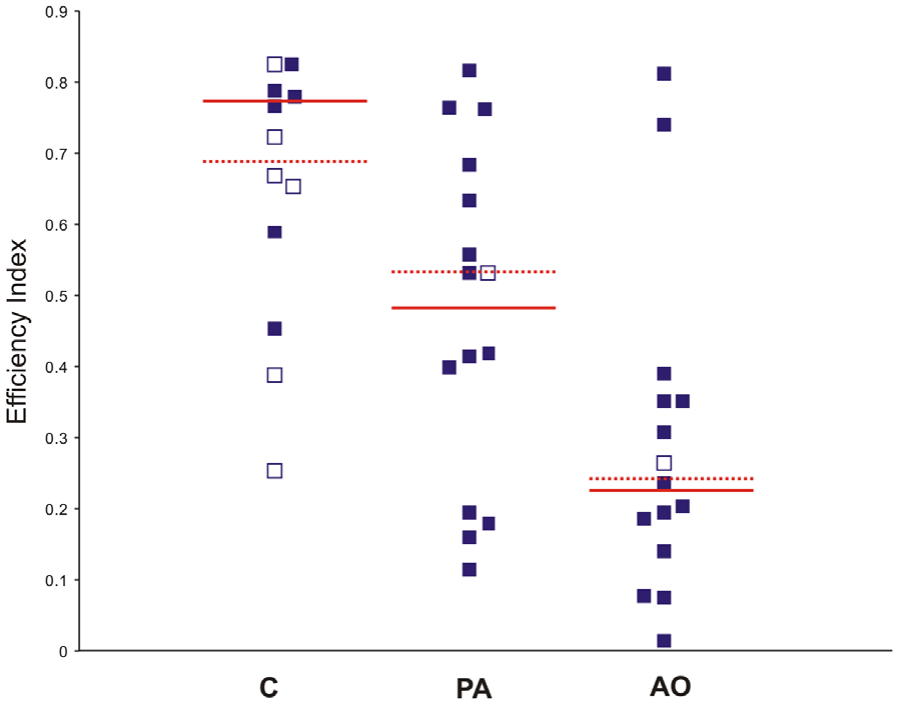
Pooled efficiency index of the section of the track recorded in the first hour after release. The filled and open symbols represent the data included and not included in the pooled analysis respectively. The median values of the data included in the pooled analysis are indicated by the unbroken lines. The dotted lines indicate the median values of the whole data set.

The statistical analysis of the pooled efficiency indexes relative to the data from both sites (Figure 10) resulted in a significant difference between groups (Kruskall-Wallis test, p<0.05). In particular, the control group displayed an efficiency index significantly higher with respect to the AO birds (Dunn's test, C vs AO p<0.01). No differences emerged between the PA pigeons and both the other two experimental groups.

### Tortuosity and homeward component

**Figure 11:**
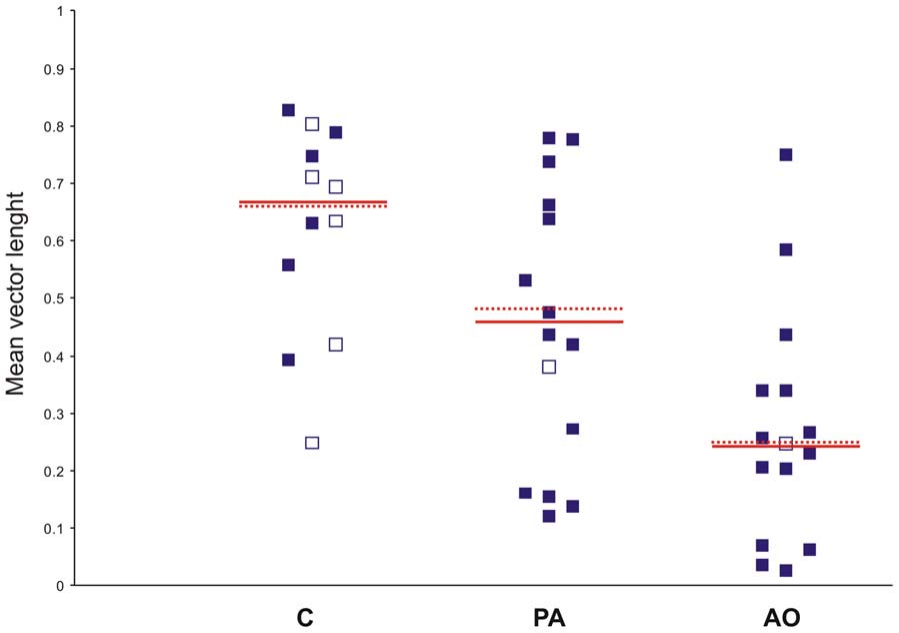
Tortuosity of the sections of the tracks recorded in the first hour after release, expressed by the mean vector lengths (see Materials and methods for further explanations). Other explanations as in Figure 10.

**Figure 12:**
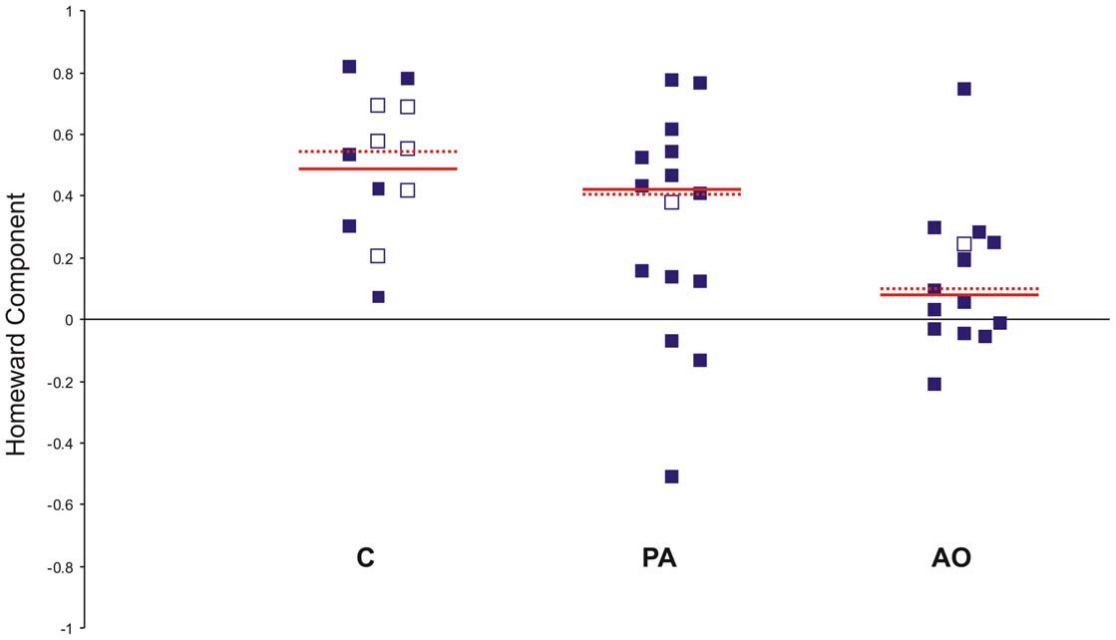
Homeward component of the sections of the tracks recorded in the first hour after release. Other explanations as in Figure 10.

The statistical analysis of tortuosity (Fig 11) and homeward component (Figure 12), done on the pooled data from both sites, revealed a significant difference between the groups (Kruskall-Wallis test: tortuosity p<0.01; homeward component p<0.05). In particular the AO pigeons displayed a more tortuous path (Dunn's test p<0.01, median = 0.24) and a lower homeward component (p<0.05, median = +0.08) than the C pigeons (median tortuosity = 0.69, median hc = .+0.48). The PA birds were not significantly different from the other two groups both in tortuosity (median = 0.46) and homeward component (median = +0.42).

### Flight time

The statistical analysis on the percentage of time the birds spent flying during the first hour from release did not highlight any difference between groups (Kruskall-Wallis test, Bolgheri p = 0.054; Montespertoli p>0.05) although the AO pigeons tended to stop longer (median percentage of time in flight: Bolgheri C 80%, PA 48%, AO 29%; Montespertoli C 65%, PA 52%, AO 18%).

### Virtual vanishing bearings

**Figure 13:**
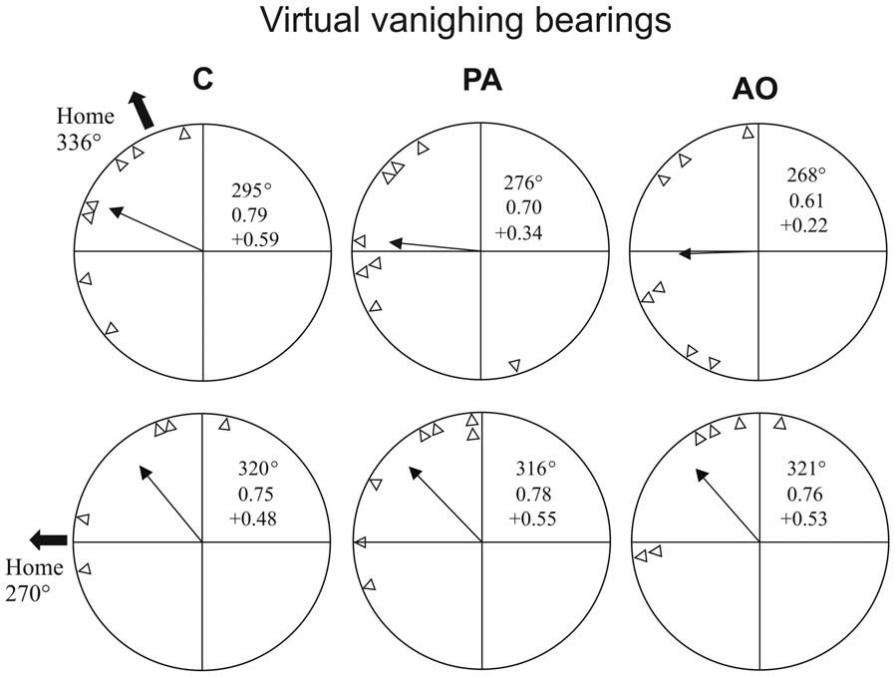
Virtual vanishing bearings. Triangles inside the diagram represent the orientation of each bird at 2 km from the release site. Outer arrows: home direction. Inner arrows: mean vector of the distribution. Mean vector length and direction, and homeward component are reported (see Results for further explanations).

The virtual vanishing bearing distributions are shown in Figure 13. At Bolgheri the virtual vanishing bearings distribution of the C pigeons (mean vector length and direction: n = 7 r = 0.79 α = 295° hc = +0.59) was significantly different from random both according to the Rayleigh (p<0.01) and V test (p<0.05). The PA group displayed a virtual vanishing bearing distribution (n = 8 r = 0.70 α = 276° hc = +0.34) different from random according to the Rayleigh test (p<0.02), but not according to the V test (p>0.05), which takes into account the orientation towards the expected direction. The AO pigeons displayed an impaired initial orientation performance (n = 7 r = 0.61 α = 268° hc = +0.22) as their virtual vanishing distribution was not different from random according to both the Rayleigh and V test (p>0.05). At Montespertoli the C pigeons' virtual vanishing bearing distribution (n = 5 r = 0.74 α = 320° hc = +0.48) was not significantly different from random both according to the Rayleigh (p = 0.054) and V test (p>0.05). At this release site both PA (n = 7 r = 0.78 α = 316° hc = +0.55) and AO pigeons (n = 6 r = 0.76 α = 321° hc = +0.50) displayed significantly oriented virtual vanishing bearing distributions (Rayleigh test: p<0.01, V test: p<0.05).

### Homing performance

**Figure 14:**
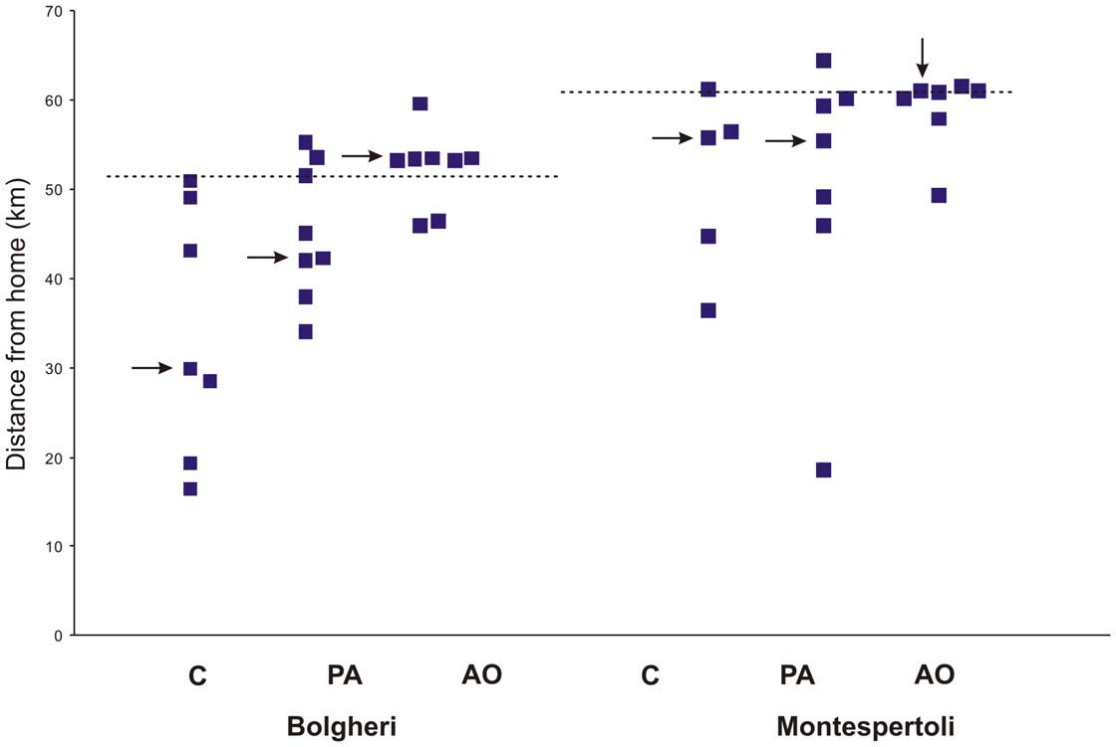
Distance from home. Distance of the birds from home recorded 1 hour after release. Broken line: distance of the release site from home. The arrows indicate the median value of the group.

Considering the last position of the birds recorded within the first hour (Figure 14), the Kruskall Wallis test revealed a significant difference in the distance from home between the groups (p = 0.012) in the release from Bolgheri (home distance 53 km). In particular the C birds were significantly closer to home than the AO pigeons (Dunn's test p<0.01; median distance from home in km: C 29.9; PA 43.8, AO 53.5). In the release from Montespertoli the GPS of the C bird #cgr stopped recording after 20 minutes from release. Therefore we considered in the analysis the last recorded position in the calculation of the distance from home. The distance from home in the first hour from release did not differ significantly (Kruskall Wallis p = 0.187) among the birds released at Montespertoli (home distance 61 km), although the same trend in the values of the median distance from home occurred (C 55.9; PA 55,5; AO 61). Looking at the individual performances, it is worth noting that the AO birds were consistently in the vicinity of the release site for both release experiments one hour after release. The three groups of pigeons were significantly different in their homing performances when released at Bolgheri (Kruskall-Wallis test, p<0.005). In fact, the C pigeons (median homing speed 7.97 km/h) were significantly faster at homing than both PA (half of the PA birds homed later than the day of release; Dunn's test: C vs PA p<0.01) and AO (more than half of the AO birds homed the day after the test; Dunn's test C vs AO p<0.05). In contrast, no statistical difference between groups emerged at Montespertoli (more than half of both the C and PA birds homed within the next day of the test; more than half of the AO birds did not home or homed later than the day after the test).

The use of the GPS logger that allowed for remote downloading from a plane avoided the selection of the best performing subjects to be included in the analysis and gave us information about the behaviour of non-homed birds. In the case of the three lost control pigeons we were able to document at least the initial track of one of them (#cgr) and the position of another subject which was localised near the home area (#cm) after a few hours from release. The lost PA pigeon (#vbcb) oriented towards north in the first two hours after release, while in the third hour corrected its orientation towards the home direction. All the lost AO birds (#mv, #mr, #rgcg) remained near the release site even in the third hour after release.

## Discussion

The olfactory navigation hypothesis attributes a specific role of olfactory environmental cues in pigeons' navigation. On the contrary, the olfactory activation hypothesis explains the impaired performances of anosmic pigeons largely documented by experiments conducted over forty years (see [2]) by assuming that odour perception is needed to activate a navigational mechanism based on non-olfactory cues. According to the olfactory activation hypothesis, pigeons prevented from smelling environmental odours during transportation, and released under nasal anaesthesia should display unimpaired navigational performances, provided that they had been stimulated by artificial odours before release. The data reported in this experiment are consistent with the olfactory navigation hypothesis and contradict the olfactory activation hypothesis.

As the effect of nasal anaesthesia decreases over time, the analysis of movement behaviour restricted to the first hour after release is the most informative about the effects of the olfactory experience prior to release. Considering the sections of the tracks recorded during the first hour of flight, it emerges that the pigeons exposed to artificial odours before being subjected to nasal anaesthesia and then released, displayed tortuous flight paths with poor homeward orientation. This was similar to what we observed for the birds transported in pure air without additional olfactory stimulation. The behaviour of both the PA and, more importantly, the AO groups clearly differs from that of the control pigeons, which were exposed to environmental olfactory information during transportation and at the release site before receiving nasal anaesthesia. In fact, the C birds displayed significantly oriented mean vector distributions at both sites, with a second order mean vector direction close to the home direction; the PA pigeons' mean vector distributions were not different from random at both sites and the AO pigeons displayed a scattered mean vector distribution at Bolgheri, and a significantly oriented distribution at Montespertoli, but with a small homeward component. In addition, the birds stimulated with artificial odours turned out to circle in the release site area without gaining distance from it. In fact, their efficiency index, calculated on the section of the track recorded on the first hour after release, was significantly lower in comparison to that of the control pigeons. Also this aspect of their behaviour contradicts the expectations predicted by the olfactory activation hypothesis.

On the whole, the PA pigeons tended to show performances intermediate between those of the control birds and the birds stimulated with artificial odours for all the parameters considered. A visual inspection of the tracks revealed that some of the PA pigeons displayed homeward orientation already within the first hour after release. Moreover, if we consider the first two and the first three hours after release, the tendency of the PA pigeons to recover their navigational abilities over time becomes evident. In contrast, the performance of the AO pigeons turned out to be impaired also after including in the analysis the portions of the tracks recorded after the effect of anaesthesia had probably ceased. In fact, the mean vector distributions of the AO pigeons were not significantly different from random, even when considering the portions of the tracks recorded in the first two (both at Bolgheri and Montespertoli) or in the first three hours (at Bolgheri) after release. Therefore the exposition to the artificial odours seemed to have an even more dramatic effect on the abilities of the birds to head home than just the permanence in pure air. To explain this fact it is well worth discussing the efficacy of nasal anaesthesia on the olfactory sense. It was shown previously using cardiac acceleration recording in response to odour stimuli that nasal anaesthesia delivered both through the nostrils [25] or the choanae [27] largely abolished the pigeons' sensitivity to smells. However, the effect decreases over time starting from 15 minutes after the treatment, and it is quite variable across individuals also because it proved difficult to standardise the administration of the spray [25]. In particular, the treatment through choanae, as in the present paper, seems to abolish the olfactory sensitivity for about one hour. The variable and temporal efficacy of the anaesthesia of the mucosae is consistent with the pattern observed in the PA pigeons, and this might explain the good performances of some of the PA birds even in the first hour of flight. It is worth noting that the homing experiments in which birds were made anosmic with nasal anaesthesia have produced variable results, while it has been consistently demonstrated that pigeons made anosmic with long lasting methods (zinc-sulphate treatment of the olfactory mucosae or olfactory nerve section) consistently produced a dramatic navigational impairment [2]. Differently from the PA, the AO pigeons seemed to be consistently impaired and confused for a longer time than that expected on the basis of the duration of the anaesthesia. One possibility is that when the anaesthesia had ceased the AO pigeons started to smell the artificial odours that were likely to be impregnated in their feathers during their permanence in the air-tight containers. Therefore the perception of the artificial odours might have prevented the perception and/or the recognition of the environmental odours. Another possible explanation is that the stimulation with artificial odours during transportation and at the test site before the release, might have induced the birds to look for the same kind of stimuli in the environment once the olfactory perception was recovered. A third explanation is that the olfactory receptors intensively stimulated with concentrations of odours much higher than the natural environmental smells are not able to detect very diluted odours, such as those contained in the environment, for sometime. Whatever the explanation might be, our data are consistent with observations by Benvenuti et al. [20], [21], that the stimulation with artificial non-sense odours at the release site acted as a disturbing factor and induced an impairment in pigeons' initial orientation and homing times. The AO birds therefore received a double manipulation: the lack of exposition to environmental air during transportation and at the release site, and exposition to artificial odours masking and confounding the perception of the natural odours. This can plausibly explain the consistently poor navigational performances of the AO group.

The specific role of environmental odours in pigeon navigation has been demonstrated in a variety of experiments on intact birds when wind-born odours were manipulated at the home loft [2]. All these data are consistent with the olfactory navigation hypothesis, but contradict the olfactory activation hypothesis. Along this line of argument it is worthwhile discussing the overall plausibility of the olfactory activation hypothesis. Among all the sensory manipulations [2] applied on homing pigeons released at unfamiliar locations, only olfactory manipulations consistently produced a homing impairment in the treated birds. To accept the olfactory activation hypothesis one should therefore admit that pigeons are never impaired at homing after a direct manipulation of their navigational mechanism, but only after the manipulation of the system (the olfactory system) activating it. We consider this implausible and in open contrast to the principle of parsimony.

Both our current GPS tracking data including non-homing homing pigeons for the first time, as well as the vast amount of historical initial orientation evidence on the effect of olfactory manipulation in pigeons [10]–[16], [18], [20], [21], contradict the interpretation of the data reported by Jorge et al [8], [9]. We could not detect an activational role of olfactory information in pigeon navigation. The inconsistencies of Jorge et al.'s studies with previous data might partly be explained by a variability in the effect of the nasal anaesthesia, and partly by the noise inherent in the vanishing bearings data. Interestingly, in our experiment the analysis of the virtual vanishing bearings distribution did not fully correspond with the pigeons' behaviour “en route”. Specifically, the virtual vanishing bearing results were consistent with what we observed from the tracks in the release at Bolgheri, but not at Montespertoli. Nevertheless we are convinced that the observation of vanishing bearings is an important method, which becomes highly reliable and meaningful in those cases in which the initial orientation data can be interpreted considering also the homing performance of the released birds. This condition is encountered when the effect of a long lasting manipulation is tested, which is not the case when applying nasal anaesthesia. Therefore GPS tracking of both homing and non-homing individuals can be critically important for resolving controversial aspects on birds navigation [28], or for highlighting phenomena not detectable by observing the birds' initial orientation [29].

In conclusion, consistently to what was reported in previous studies, the results of our experiment constitute further evidence against the activational role of odours in birds' navigation. Instead, our data provide a new piece of evidence for a specific role of olfactory information in the navigational map mechanism in homing pigeons.
